# Intensive Management of a Patient with HIV, Active Tuberculosis, and COVID-19: A Multidisciplinary Approach in the Intensive Care Unit

**DOI:** 10.3390/life15091435

**Published:** 2025-09-13

**Authors:** Brayan Ricardo Mosquera-Arias, Valeria Sanclemente-Cardoza, Jose Luis Estela-Zape

**Affiliations:** 1 Faculty of Health, Universidad Santiago de Cali, Cali 760035, Colombia; dirmedicinacali@usc.edu.co; 2 Faculty of Health, School of Public Health, Universidad del Valle, Cali 760043, Colombia; valeriasanclemente0@gmail.com; 3 Health and Movement Research Group, Universidad Santiago de Cali, Cali 760035, Colombia

**Keywords:** HIV, tuberculosis, COVID-19, inflammation, anti-retroviral agents, antitubercular agents, critical care

## Abstract

Coinfection with HIV, active tuberculosis, and COVID-19 is rare but markedly increases mortality risk and complicates treatment due to the interactions between these infections. Management requires a multidisciplinary approach that integrates antiretroviral therapy, antituberculous drugs, antibiotics, and supportive care for COVID-19. We report the case of a 28-year-old male with HIV (viral load 30 copies, CD4 count 303), active tuberculosis, and a history of resolved syphilis, who presented with severe respiratory decompensation and hypoxemia (SpO_2_ 55%), requiring orotracheal intubation. Initial treatment included broad-spectrum antibiotics, antiretrovirals, and antituberculous therapy. Despite the critical illness, the patient demonstrated progressive clinical improvement, was successfully extubated after a spontaneous breathing trial, and continued recovery under supplemental oxygen. This case underscores the clinical complexity of triple coinfection and highlights the potential for favorable outcomes when management is timely and multidisciplinary.

## 1. Introduction

The management of patients with HIV, active tuberculosis (TB), and COVID-19 presents a significant clinical challenge due to the complex interactions between these infections. Coinfection with these three diseases is uncommon but leads to a substantial increase in morbidity and mortality [1,2,3]. HIV weakens immune function, making individuals more susceptible to opportunistic infections like TB, which remains a major cause of death among HIV-positive individuals [4]. The COVID-19 pandemic further complicates this by impairing respiratory function and modifying immune responses, which increase the risk of severe complications in coinfected patients [5].

The combination of HIV and active TB in patients with COVID-19 significantly raises mortality, with a 38% increased risk of death compared to those without these comorbidities [6]. Additionally, active TB exacerbates pulmonary dysfunction, increasing the likelihood of respiratory failure in the context of COVID-19, which complicates clinical management [7].

The management of these patients requires a multidisciplinary approach that integrates antiretroviral therapy (ART), antituberculous treatment, and appropriate COVID-19 care [8,9,10]. Therapeutic strategies must be tailored to minimize drug interactions and adverse effects. This case report outlines the intensive clinical management of a patient with HIV, active TB, and COVID-19, emphasizing the application of an integrated therapeutic approach and multidisciplinary intervention in the context of complex comorbidities. The report is presented in accordance with the SCARE 2023 guidelines [11], ensuring methodological rigor and transparency in case reporting.

## 2. Case Report

A 28-year-old male patient with a history of resolved syphilis, HIV (viral load 30 copies, CD4 count 303), and active TB (first phase of treatment initiated on 9 August 2024, with expected completion by 13 October 2024) is currently undergoing the second phase of TB treatment (biconjugate therapy, initiated on 14 October 2024).

He was presented with decompensation for his underlying condition, characterized by 5 h of respiratory distress, multiple episodes of vomiting, and stool type 4 according to the Bristol Stool Chart [12].

**Figure 1:**
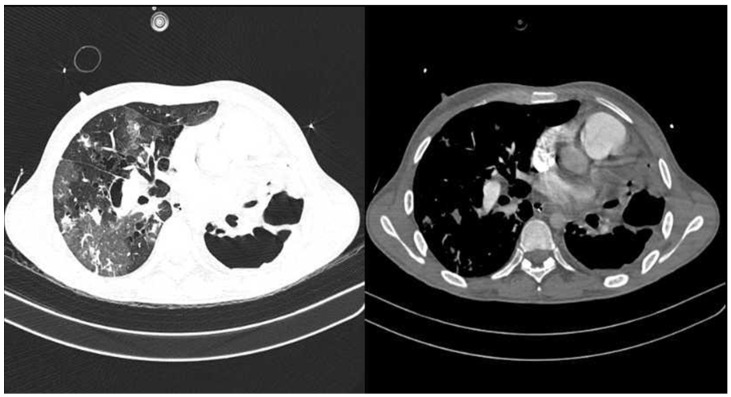
Chest angiotomography on admission. Axial contrast-enhanced chest CT images showing extensive bilateral pulmonary involvement with multiple cavitary lesions predominantly in the right lung, associated with areas of consolidation and parenchymal destruction, consistent with severe infectious processes.

**Figure 2:**
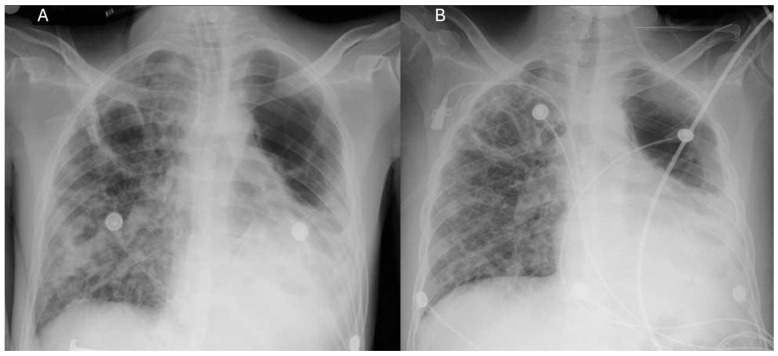
Sequential chest radiographic follow-up. (**A**) Endotracheal intubation: Multiple bullae in both lung fields, alveolar infiltrates in the right lung field, and bilateral perihilar atelectatic bands. (**B**) Post endotracheal intubation: The clouding of the left lung base persists, as do the multiple bullae in the right apex.

Upon admission, the patient exhibited progressive respiratory deterioration, utilizing accessory muscles and presenting an oxygen saturation (SpO2) of 55% despite a 50% Venturi mask, prompting orotracheal intubation. Sedation with midazolam and fentanyl, continuous cisatracurium infusion for neuromuscular blockade, and dual vasopressor support (norepinephrine and vasopressin) were required. Baseline chest imaging at the initiation of antituberculous therapy revealed left pneumothorax (~30%), multiple bilateral bullae, interstitial-alveolar infiltrates, and left basal opacification, consistent with cavitary pulmonary TB. Orotracheal intubation was performed, and diagnostic studies (Table 1) were ordered, including blood tests, a nasal swab for SARS-CoV-2 PCR-RT (Table 2), a chest angiotomography (Angio-CT) (Figure 1), and a chest X-ray (Figure 2).

In the intensive care unit (ICU), the patient developed persistent tachycardia and a tendency toward hypotension (mean arterial pressure [MAP] < 60 mmHg). Dual vasopressor support was initiated with norepinephrine (1.0 mcg/kg/min) and vasopressin (2.4 U/h). Laboratory results showed impaired oxygenation and elevated C-reactive protein (CRP), confirming the diagnosis of COVID-19 (Table 2), along with leukocytosis. Treatment with dexamethasone (6 mg/24 h) was started according to the RECOVERY protocol [13], and empirical antibiotic coverage was initiated with meropenem (2 g/8 h) and linezolid (600 mg/12 h), given the risk of secondary infections in an immunocompromised patient.

Chest imaging revealed cavities and bronchiectasis, leading to the initiation of vancomycin (1 g/12 h), liposomal amphotericin B (250 mg/24 h), and trimethoprim/sulfamethoxazole (80/400 mg/8 h). Clarification was required: The indication, duration, and discontinuation dates for each agent needed to be specified to support the therapeutic rationale. In particular, the TMP/SMX dose corresponds to prophylaxis; therefore, it must be clarified whether it was administered as prophylaxis or treatment, with the dose adjusted accordingly. These decisions were made in the absence of microbiological cultures or fungal markers, which should be reported or, alternatively, discussed within the framework of preventative strategies in profound immunosuppression.

Persistent respiratory acidosis necessitated continuous cisatracurium infusion (5 mcg/kg/min) to facilitate mechanical ventilation and maintain an RASS score of −4. Midazolam (0.05 mg/kg/h) and fentanyl (0.05 mcg/kg/h) were used for sedation and analgesia.

Antiretroviral and antituberculosis regimens were continued with emtricitabine (200 mg/4 h), tenofovir (300 mg/24 h), dolutegravir (50 mg/24 h), and rifampicin/isoniazid (4 tablets/24 h, continuation phase). It should also be clarified whether pyrazinamide and ethambutol were included during the initial intensive phase, with corresponding dates, to complete the therapeutic timeline.

After treatment adjustment, oxygenation and hemodynamic status improved, allowing the discontinuation of vasopressin, cisatracurium, and midazolam, with norepinephrine reduced to 0.06 mcg/kg/min. Propofol (1–4 mg/kg/h) was introduced to assess extubation readiness. A 120 min spontaneous breathing trial with a negative inspiratory force (NIF) of −40 cmH_2_O confirmed readiness, and the patient was successfully extubated to a 35% Venturi mask. Persistent hypoxemia (PaO_2_/FiO_2_ < 150) required escalation to high-flow nasal cannula (HFNC) at 50%, followed by de-escalation to low-flow nasal cannula at 3 L/min and subsequent discontinuation.

Auscultation revealed bilateral vesicular sounds with apical rhonchi, leading to the addition of ipratropium bromide (3 inhalations every 6 h). With subsequent improvement, oxygen support was progressively weaned from HFNC to nasal cannula at 3 L/min, which was later discontinued, and the patient was transferred to the general ward once oxygenation parameters stabilized.

## 3. Discussion

Coinfection with HIV, active TB, and COVID-19 is rare but markedly increases morbidity and mortality. This overlap presents major diagnostic and therapeutic challenges because of similar clinical features and complex drug interactions. HIV-related immunosuppression increases susceptibility to TB and complicates both the diagnosis and treatment decisions. The overlapping symptoms of HIV and TB often lead to diagnostic delays and inadequate management. In addition, immune dysfunction in HIV can result in false-negative TB tests, emphasizing the need for more sensitive diagnostic methods, such as molecular assays, for early detection [14].

Coinfection with COVID-19 further complicates the scenario, as respiratory manifestations may obscure TB-related findings, hinder clinical evaluation, and increase the risk of severe complications [15]. Early and accurate diagnosis is therefore essential to initiate timely therapy that adequately addresses all three infections. TB treatment also requires adjustment to account for interactions with ART. For example, rifampicin reduces the efficacy of several ART agents, making dose modification or substitution with alternatives such as rifabutin necessary [9,14,16,17].

Patients coinfected with HIV and TB who initiate antiretroviral therapy (ART) remain at significant risk of immune reconstitution inflammatory syndrome (IRIS). This immunopathological response, triggered by ART-induced immune recovery, can unmask or worsen TB, contributing to morbidity and complicating treatment. Consequently, close clinical surveillance is necessary to enable early recognition and timely therapeutic adjustments that mitigate its impact [2,3].

Management of HIV–TB coinfection also requires careful coordination to minimize pharmacological interactions. Rifampicin, an essential component of TB therapy, induces hepatic metabolism of several antiretroviral agents, reducing their plasma concentrations and therapeutic efficacy. In such cases, either dose modification or substitution with rifabutin becomes necessary to maintain virological suppression while preserving TB treatment effectiveness [17]. These adjustments are central to preventing opportunistic infections and avoiding therapeutic failure.

The timing of ART initiation in active TB remains a critical clinical decision. Current guidelines recommend initiating ART within two weeks for patients with CD4 counts below 50 cells/mm^3^, yet this must be balanced against the heightened risk of IRIS. Thus, treatment decisions require individualized assessment, considering both the benefits of early immune recovery and the potential complications arising from immune dysregulation [18,19].

The coexistence of COVID-19 further amplifies these challenges. Overlapping respiratory manifestations may accelerate pulmonary compromise, especially in immunocompromised hosts, increasing the risk of acute respiratory failure. In such scenarios, therapeutic strategies must remain flexible, integrating ART, antituberculous therapy, and COVID-19 management while anticipating the need for advanced respiratory support, including invasive ventilation [5,7].

Mortality in patients coinfected with HIV and TB is significantly higher when associated with COVID-19. Recent studies [3,4,6,9,20] indicate that TB may exacerbate the severity and mortality of COVID-19, emphasizing the need for the integrated management of these concurrent infections. In such cases, broad-spectrum antibiotics such as vancomycin, antifungals like liposomal amphotericin B, and antibacterial agents such as trimethoprim-sulfamethoxazole are important to prevent secondary infections, which are frequent in immunocompromised patients [4,9,14,18] and may contribute to additional pulmonary complications and increased mortality.

ART was continued with emtricitabine (200 mg once daily), tenofovir disoproxil fumarate (300 mg once daily), and dolutegravir (50 mg once daily), with dosing adjustments for rifampicin co-administration. Active TB was managed with an intensive phase of quadruple therapy including isoniazid (300 mg/day), rifampicin (600 mg/day), pyrazinamide (1500 mg/day), and ethambutol (1200 mg/day) for two months, followed by a continuation phase with isoniazid (300 mg/day) and rifampicin (600 mg/day). Adjunctive corticosteroid therapy was administered in the setting of extensive cavitary TB, recognizing that outcomes in immunocompromised patients are variable and may only partially mitigate inflammatory complications [14,20].

Managing respiratory failure was a central component of treatment. The patient developed severe respiratory decompensation that required intubation and invasive mechanical ventilation. Sedation with midazolam and analgesia with fentanyl were combined with continuous cisatracurium infusion to facilitate protective ventilation. Ventilatory management included high oxygen requirements (FiO_2_ 70%) and PEEP of 10–12 cm H_2_O during the initial phase, with plateau pressures maintained at <30 cmH_2_O. Gasometric analysis on admission confirmed severe acidosis and hypoxemia (PaO_2_/FiO_2_ 127), with gradual improvement under these settings. Dexamethasone, administered according to the RECOVERY protocol [12], contributed to the control of COVID-19-related pulmonary inflammation, though its immunosuppressive effect may have interfered with the immune response to tuberculosis. Antiviral therapy with remdesivir was not initiated because the patient did not meet the eligibility criteria. Prophylactic anticoagulation with low-molecular-weight heparin, adjusted to body weight and renal function, was maintained to reduce the risk of thromboembolic events.

Intensive monitoring of oxygenation, along with high-flow oxygen therapy, was essential to maintain respiratory stability. After a period of intubation and management of respiratory failure, the patient was successfully extubated and transitioned to high-flow nasal cannula oxygen therapy [21]. Although mild hypoxemia persisted, proper adjustment of oxygen therapy facilitated improved spontaneous breathing and supported recovery [13].

This case also emphasizes the importance of resource-appropriate antimicrobial stewardship and infection-control measures, particularly in the context of HIV, TB, and COVID-19. Rational antimicrobial use and standardized infection-prevention protocols are essential to minimize resistance, avoid unnecessary exposure, and optimize outcomes in resource-limited settings.

### New Insights for Clinical Practice

The early implementation of rapid molecular diagnostics facilitates timely identification of coinfections and accelerates therapeutic intervention. Individualized adjustment of ART in the context of rifampicin co-administration reflects the evolving need for dynamic optimization of pharmacological regimens to minimize interactions and complications. In cases of severe respiratory failure, flexible ventilatory strategies and meticulous corticosteroid titration emerge as key elements to balance the benefits of inflammation control in COVID-19 with the risks of TB progression. Furthermore, the coordination of multidisciplinary teams and the promotion of infection-control measures remain essential pillars for effective care, particularly in settings with constrained resources [15,16,19].

## 4. Conclusions

Intensive management of a patient with HIV, active TB, and COVID-19 requires a multidisciplinary approach, prompt diagnosis, coordinated treatment to manage drug interactions, and close monitoring for complications such as respiratory failure and IRIS to optimize outcomes and reduce mortality.

## Figures and Tables

**Table 1:** Longitudinal biochemical assessment during emergencies, hospitalization, and intensive care.

Laboratory Parameters	Patient’s Values						
	Emergencies Date of Entry	ICU					Hospitalization
	15 December 24	15 December 24	17 December 24	21 December 24	22 December 24	26 December 24	4 January 25
Hematology							
Hemoglobin (13.5–17.5 g/dL)	10.5	11.3	10.3	9.2	8.9	9.4	11.9
Hematocrit (40–50%)	35%	39%	36%	31%	29%	32%	40%
Platelets (150–450 × 10^3^/µL)	237	372	248	242	210	234	307
Leukocytes (4.5–11.0 × 10^3^/µL)	22.0	22.0	12.0	4.2	3.2	3.6	5.6
C-reactive protein (less than 2 mg/L)	39.5						
Lactate (0.5–2.2 mmol/L)	1.4	1.3	2.9	1.8	1.2		
Renal Function							
Creatinine (0.7–1.3 mg/dL)		1.1	0.43	0.45	0.52	0.50	0.69
Blood urea nitrogen (7–20 mg/dL)		13.5	5.9	4.2	6.8	12.1	13.4
Coagulation							
Prothrombin time (11.7–15.5 s)		12.6					
Partial thromboplastin time (24–45 s)		36.1					
Biochemistry							
Electrolytes							
Sodium (135–145 mmol/L)	137	136	126	141	142	138	
Potassium (3.5–4.5 mmol/L)	5.1	5.4	4,9	3.4	3.4	3.6	3.9
Chloride (95–105 mmol/L)	105.8	102.0	99.9	106.2	108.7	100.9	
Arterial blood gases, mmHg							
Supplemental oxygen support	Venturi mask	Invasive mechanical ventilation			High-flow nasal cannula	Simple nasal cannula	Ambient breathing
FiO_2_	50%	70%	40%	50%	50%	28%	21%
Flow per minute					50 L/min		
pH (7.35–7.45)	6.93 (pre-intubation)	7.10	7.14	7.39	7.40	7.41	7.30
pCO_2_ (35–45 mmHg)	113.0	72	68	44	44.0	46	46
PaO_2_ (75–100 mmHg)	128.0	89	97	93	158	87	66
HCO_3_^−^ (22–26 mmol/L)	15.1	22.4	23.1	26.6	27	29.2	22
BE (–2 to +2 mmol/L)	−12.5	−7.4	−6.6	1.5	2.2	4.0	−3.8
PaO_2_/FiO_2_ > 400 (ratio)	256	127	242	186	316	310	314
ROX Index (SpO_2_/FiO_2_)/RR					15 (indicative of high probability of HFNC success, threshold > 5)		

**Table 2:** Microbiological and virological results.

Test	Sample	Result	Interpretation
Respiratory panel	Nasopharyngeal swab	Negative	No respiratory viral or bacterial pathogens detected
SARS-CoV-2 RT-PCR	Nasopharyngeal swab	Positive	Confirmed COVID-19 infection
TB drug susceptibility (DST)	Sputum (SOT)	Sensitive pattern	Sensitive pattern
Blood culture 1	Blood	Negative	No bacterial growth
Blood culture 2	Blood	Negative	No bacterial growth

## Data Availability

The authors declare that all data supporting the report are available upon request from the corresponding author.
